# Hemophagocytic Lymphohistiocytosis Secondary to Immunotherapy Toxicity in a Patient With Breast Cancer: A Case Report

**DOI:** 10.7759/cureus.101206

**Published:** 2026-01-09

**Authors:** Abdullah Alhouri, Hari Johal, Ricky Frazer

**Affiliations:** 1 Internal Medicine, Aneurin Bevan University Health Board, Newport, GBR; 2 Cardiology, University Hospital of Wales, Cardiff, GBR; 3 Immuno-Oncology, Velindre Cancer Center, Cardiff, GBR

**Keywords:** anti-pd-1, hemophagocytic lymphohistiocytosis (hlh), immune checkpoint inhibitor, immunotherapy-related adverse events, pembrolizumab

## Abstract

Hemophagocytic lymphohistiocytosis (HLH) is a rare and potentially fatal hyperinflammatory syndrome marked by abnormal activation of the immune system, resulting in widespread tissue destruction and multi-organ failure. HLH can be classified as primary (genetic) or secondary, with triggers including infections, malignancies, autoimmune diseases, and increasingly, immunotherapy drugs. Early diagnosis is difficult because the clinical presentation is frequently nonspecific, including hepatosplenomegaly, cytopenias, persistent fever, and elevated inflammatory markers, and diagnosis is typically guided by established frameworks such as the Hemophagocytic Lymphohistiocytosis-2004 (HLH-2004) criteria or the H-score.

We describe a case of HLH in a patient with breast cancer whose HLH developed after administration of pembrolizumab, a programmed cell death protein-1 (PD-1) inhibitor, used to treat her cancer. The patient developed multi-organ inflammation, cytopenias, and constitutional symptoms, initially confounded with other toxicities of antitumour therapy, which impeded prompt diagnosis. Laboratory and histopathological findings ultimately supported the diagnosis of HLH.

This report emphasizes the need for clinician awareness of HLH as a rare but serious immune-related adverse event (irAE) in the context of cancer immunotherapy.

## Introduction

Hemophagocytic lymphohistiocytosis (HLH) is a syndrome characterized by uncontrolled immune activation, leading to a hyperinflammatory state, a cytokine storm, and multi-organ failure [1]. It was first described in pediatric populations as a genetic disorder, now referred to as primary HLH, but is now widely acknowledged in adults as a secondary phenomenon triggered by infections, cancers, autoimmune diseases, or treatments such as immunotherapy inhibitors [2,3].

Clinically, HLH is characterized by persistent fever, cytopenias, hepatosplenomegaly, hyperferritinemia, and features of systemic inflammation. The condition is associated with high mortality rates, estimated between 20% and 40%, depending on the severity at presentation and time to treatment initiation [4,5]. The Hemophagocytic Lymphohistiocytosis-2004 (HLH-2004) diagnostic criteria and the H-score remain the most widely used diagnostic frameworks, although distinguishing HLH from severe sepsis or malignancy-associated inflammation remains challenging [3].

The introduction of immune checkpoint inhibitors (ICIs), such as anti-programmed cell death protein 1 (anti-PD-1) agents (pembrolizumab, nivolumab), anti-programmed death-ligand 1 (anti-PD-L1) agents, and anti-cytotoxic T-lymphocyte-associated antigen 4 (anti-CTLA-4) agents, has revolutionized cancer therapy. These drugs work by blocking inhibitory signals on T cells, thereby promoting durable antitumor immune responses [4]. However, this mechanism also predisposes patients to immune-related adverse events (irAEs), which can affect any organ system. Common irAEs include thyroiditis, colitis, hepatitis, and pneumonitis [6]. Rarely, fulminant hyperinflammatory syndromes such as HLH can occur [6].

Understanding atypical and potentially fatal immune toxicities is essential, given the extensive use of ICIs in solid tumors such as breast cancer. Due to the limited number of case reports and small series on ICI-induced HLH, clinical diagnosis and treatment remain extremely difficult. This case highlights the emerging association between ICIs and HLH, emphasizing the importance of maintaining a high index of suspicion for this hyperinflammatory condition in patients receiving immunotherapy. Early recognition and prompt initiation of targeted immunosuppressive therapy are critical to improving outcomes.

## Case presentation

A 57-year-old woman with locally advanced breast cancer was admitted following acute clinical deterioration. Between October 2024 and March 2025, she completed eight cycles of neoadjuvant chemoimmunotherapy, consisting of pembrolizumab (eight cycles) and carboplatin and paclitaxel (four cycles, October-December 2024), followed by epirubicin and cyclophosphamide (four cycles, January-March 2025). She demonstrated an excellent tumor response, and curative-intent surgery was scheduled for April 2025.

Her past medical history included chronic lymphocytic leukemia, managed conservatively under surveillance; rheumatoid arthritis, previously treated with methotrexate but discontinued six years earlier; and hypothyroidism secondary to immunotherapy, for which she had been maintained on thyroxine since December 2024. She had no history of hemophagocytic syndromes or familial immune disorders.

She presented four weeks following completing neoadjuvant chemoimmunotherapy with an acute onset of profound hypotension, tachycardia, and peripheral shutdown, requiring urgent admission to the intensive care unit. On examination, she was febrile (38.9 °C), hypotensive (systolic blood pressure 70 mmHg), and had evidence of multi-organ dysfunction. Initial laboratory studies revealed pancytopenia, with a hemoglobin level of 8.6 g/dL, a white cell count of 1.4 × 10^9/L, and a platelet count of 65 × 10^9/L. Coagulation studies revealed derangement consistent with disseminated intravascular coagulation (DIC): prothrombin time (PT), 19 seconds; fibrinogen, 0.8 g/L; and markedly elevated D-dimer (>20,000 ng/mL).

Biochemical analyses demonstrated acute kidney injury with creatinine rising to 265 µmol/L (baseline 85 µmol/L) and urea 17 mmol/L. Inflammatory markers were significantly elevated, including ferritin >15,000 µg/L, triglycerides 4.8 mmol/L, and lactate dehydrogenase (LDH) 1534 U/L. C-reactive protein (CRP) was elevated at 285 mg/L, while procalcitonin was modestly raised at 1.2 µg/L. Liver function tests showed mild transaminitis (alanine aminotransferase (ALT) 87 U/L, aspartate aminotransferase (AST) 30 U/L). Viral reactivation, including Epstein-Barr virus (EBV) and cytomegalovirus (CMV), was negative. The bone marrow aspirate is a largely acellular sample, so it may not be representative; however, the constellation of features met the HLH-2004 diagnostic criteria [3].

She required escalating organ support, including high-dose noradrenaline (0.8 mg/hour) and continuous venovenous hemofiltration for acute kidney injury. Broad-spectrum antimicrobials were administered empirically, including meropenem, clindamycin, and vancomycin. Given a weakly positive beta-D-glucan, antifungal therapy with posaconazole was initiated but discontinued after further evaluation showed no evidence of fungal disease.

Despite maximal supportive measures, the patient’s hyperinflammatory state persisted, characterized by progressive cytopenias and worsening coagulopathy. A diagnosis of HLH was established based on fulfillment of at least six HLH-2004 criteria: fever, cytopenia affecting ≥2 lineages, hyperferritinemia, hypofibrinogenemia, hypertriglyceridemia, and evidence of coagulopathy and multi-organ dysfunction.

In the absence of an infectious or malignant trigger, and given her recent exposure to pembrolizumab, the HLH was attributed to ICI-induced immune dysregulation. The patient was commenced on intravenous methylprednisolone, followed by anakinra 200 mg three times daily, with gradual tapering and eventual dose reduction to 100 mg as her clinical and biochemical parameters improved (Table 1).

**Table 1:** A summary of the evolution of hematological and biochemical parameters in relation to the initiation and tapering of methylprednisolone and anakinra therapy. TDS: three times daily

Parameter	Normal Reference Range	Admission	Started on Methylprednisolone	Started on Anakinra 200 mg TDS	Five Days After Starting Anakinra	After Completing Seven Days of Anakinra 200 mg TDS	Anakinra Reduced to 100 mg	Started Weaning Prednisolone	One Month Post Admission	Two Months Post Admission
Hemoglobin (g/dL)	12.0–15.5	8.6	8.5	7.6	6.3	6.3	7.3	8.6	8.9	10.7
White Cell Count (×10⁹/L)	4.0–11.0	1.4	3.6	10.3	1.6	1.9	2.1	2.1	2.7	5.9
Neutrophils (×10⁹/L)	2.0–7.5	3.1	1.2	2.6	0.7	0.9	1.1	1.2	1.7	5.2
Platelets (×10⁹/L)	150–400	65	61	70	11	22	37	79	110	140
Triglycerides (mmol/L)	<1.7	-	4.6	6.1	5.2	4.7	2.1	2.4	2.3	1.6
Lactate Dehydrogenase, LDH (U/L)	120–250	-	-	1534	-	448	-	713	682	269
Aspartate Aminotransferase, AST (U/L)	10–40	-	30	-	27	33	-	24	24	22
Ferritin (µg/L)	15–200	149	883	>15,000	4074	1350	1183	1209	1333	966
Alkaline Phosphatase, ALP (U/L)	40–120	15	142	573	576	455	458	302	257	63
Alanine Aminotransferase, ALT (U/L)	7–56	11	14	87	28	32	37	27	27	25
Bilirubin (µmol/L)	<21	4.9	1.4	0.9	0.7	1.1	1.2	1.4	1.5	2.4
Fibrinogen (g/L)	2.0–4.0	0.8	1.4	0.9	0.7	1.1	1.2	1.4	1.5	2.4

## Discussion

HLH is a hyperinflammatory syndrome characterized by uncontrolled immune activation and cytokine storm, leading to multi-organ dysfunction and high mortality if untreated [1,4,5]. In adults, HLH is most often secondary to malignancy, infection, or autoimmune disease [2]. The growing use of ICIs has led to iatrogenic HLH being recognized as a rare but increasingly recognized irAE.

Epidemiology and previous reports

A recent systematic review identified 51 published cases of ICI-induced HLH since 2017, with an overall mortality rate of 13.7% [7]. More than half of patients received corticosteroids alone, but a smaller percentage needed etoposide or cytokine-targeted treatment, particularly tocilizumab, which was associated with a 100% survival rate [7].

Several case reports have specifically linked pembrolizumab to HLH across different cancer types. For example, patients with head and neck squamous cell carcinoma, lung adenocarcinoma, and melanoma all developed HLH during pembrolizumab treatment [8, 9]. Responses to treatment varied from corticosteroid monotherapy to escalation with etoposide or pulse methylprednisolone [8]. Notably, a recent report documented HLH in a triple-negative breast cancer patient receiving neoadjuvant pembrolizumab, which was successfully managed with dexamethasone monotherapy [10]. Our case, therefore, represents one of the very few reported instances of pembrolizumab-associated HLH in breast cancer, adding to this limited but clinically important literature. 

Pathophysiology

ICIs function by releasing inhibitory checkpoints on T cells, thereby restoring antitumor immunity. However, this can also result in uncontrolled T-cell activation, cytokine release (e.g., interferon-gamma (IFN-γ), tumor necrosis factor-alpha (TNF-α), interleukin-6 (IL-6)), and macrophage hyperactivation. PD-1 blockade in particular has been implicated in promoting macrophage activity, which may exacerbate hemophagocytosis [11]. This dual mechanism of cytotoxic T-cell and macrophage overactivation underpins the immunopathology of HLH in the context of ICIs.

Diagnostic challenges

The diagnosis of HLH remains difficult because its clinical presentation overlaps substantially with sepsis, DIC, and chemotherapy-related toxicities. In critically ill patients, features such as persistent cytopenias, hyperferritinemia, and hepatosplenomegaly should raise suspicion for HLH, particularly in the absence of an identifiable infectious source [3]. The HLH-2004 criteria and H-score remain the most widely applied diagnostic tools in this setting [3].

Therapeutic considerations

Management of ICI-induced HLH centers on withdrawal of immunotherapy and initiation of immunosuppression. Corticosteroids are typically first-line and have demonstrated efficacy in numerous reports [8,9,12]. Escalation with etoposide has been employed in refractory cases, while cytokine-directed therapies such as tocilizumab have shown promise in mitigating hyperinflammation [7, 8,11,12]. Importantly, IL-6 blockade appears particularly beneficial in patients with markedly elevated inflammatory markers, suggesting a potential role for biomarker-guided therapy.

## Conclusions

This case highlights the need for heightened vigilance among oncologists and intensivists for HLH in patients presenting with unexplained shock, cytopenia, and systemic inflammation following ICI therapy. Early differentiation from sepsis is critical, as delayed recognition significantly worsens outcomes. Multidisciplinary involvement, spanning oncology, hematology, immunology, and intensive care, is essential.

Furthermore, the recurrence of HLH across diverse cancers and ICI regimens suggests a shared immunopathological mechanism. Future research should focus on developing consensus treatment algorithms, evaluating the role of cytokine-directed therapy, and identifying predictive biomarkers or genetic predispositions that may allow earlier recognition of high-risk patients.

## References

[1] La Rosée P, Horne A, Hines M (2019). Recommendations for the management of hemophagocytic lymphohistiocytosis in adults. Blood.

[2] Janka GE (2012). Familial and acquired hemophagocytic lymphohistiocytosis. Annu Rev Med.

[3] Henter JI, Horne A, Aricó M (2007). HLH-2004: diagnostic and therapeutic guidelines for hemophagocytic lymphohistiocytosis. Pediatr Blood Cancer.

[4] Noseda R, Bertoli R, Müller L, Ceschi A (2019). Haemophagocytic lymphohistiocytosis in patients treated with immune checkpoint inhibitors: analysis of WHO global database of individual case safety reports. J Immunother Cancer.

[5] Kenworthy C, Di M, Deshpande H (2022). Hemophagocytic lymphohistiocytosis as a complication of immune checkpoint inhibitor therapy for sarcoma. Curr Probl Cancer Case Rep.

[6] Mitchell JA, Gillam EM, Stanley LA, Sim E (1990). Immunotoxic side-effects of drug therapy. Drug Saf.

[7] Walmsley CS, Schoepflin Z, De Brabandt C, Rangachari D, Berwick S, Patell R (2025). Hemophagocytic lymphohistiocytosis associated with immune checkpoint inhibitor use: a review of the current knowledge and future directions. Blood Cells Mol Dis.

[8] Kalmuk J, Puchalla J, Feng G, Giri A, Kaczmar J (2020). Pembrolizumab-induced hemophagocytic lymphohistiocytosis: an immunotherapeutic challenge. Cancers Head Neck.

[9] Zhai C, Jin X, You L (2024). Hemophagocytic lymphohistiocytosis following pembrolizumab and bevacizumab combination therapy for cervical cancer: a case report and systematic review. BMC Geriatr.

[10] Patton L, Monteith B, Heffernan P, Herzinger T, Wilson BE (2024). Hemophagocytic lymphohistiocytosis/cytokine release syndrome secondary to neoadjuvant pembrolizumab for triple-negative breast cancer: a case study. Front Oncol.

[11] Dupré A, Michot JM, Schoeffler A (2020). Haemophagocytic lymphohistiocytosis associated with immune checkpoint inhibitors: a descriptive case study and literature review. Br J Haematol.

[12] (2026). Management of immunotherapy-related toxicities. https://www.geoq.info/pub/documents/IMMUNOTHERAPIE/immunotherapie_lignes-directrices_238.pdf?utm_source=chatgpt.com.

